# Rubidium-82 generator yield and efficiency for PET perfusion imaging: Comparison of two clinical systems

**DOI:** 10.1007/s12350-020-02200-6

**Published:** 2020-05-20

**Authors:** Ali Ahmadi, Ran Klein, Howard C. Lewin, Rob S. B. Beanlands, Robert A. deKemp

**Affiliations:** 1 Division of Cardiology, University of Ottawa Heart Institute, National Cardiac PET Centre, 40 Ruskin Street, Ottawa, ON K1Y 4W7 Canada; 2 Division of Nuclear Medicine, The Ottawa Hospital, Ottawa, ON Canada; 3 Cardiac Imaging Nuclear Associates, Los Angeles, CA USA

**Keywords:** Myocardial perfusion imaging, positron emission tomography, rubidium-82 generator, daily quality assurance

## Abstract

**Introduction:**

Strontium-82/Rubidium-82 (^82^Sr/^82^Rb) generators are used widely for positron emission tomography (PET) imaging of myocardial perfusion. In this study, the ^82^Rb isotope yield and production efficiency of two FDA-approved ^82^Sr/^82^Rb generators were compared.

**Methods:**

N = 515 sequential daily quality assurance (QA) reports from 9 CardioGen-82^®^ and 9 RUBY-FILL^®^ generators were reviewed over a period of 2 years. A series of test elutions was performed at different flow-rates on the RUBY-FILL^®^ system to determine an empirical correction-factor used to convert CardioGen-82^®^ daily QA values of ^82^Rb activity (dose-calibrator ‘maximum’ of 50 mL elution at 50 mL·min^−1^) to RUBY-FILL^®^ equivalent values (integrated ‘total’ of 35 mL elution at 20 mL·min^−1^). The generator yield (^82^Rb) and production efficiency (^82^Rb yield/^82^Sr parent activity) were measured and compared after this conversion to a common scale.

**Results:**

At the start of clinical use, the system reported ^82^Rb activity from daily QA was lower for CardioGen-82^®^ vs RUBY-FILL^®^ (2.3 ± 0.2 vs 3.0 ± 0.2 GBq, *P* < 0.001) despite having similar ^82^Sr activity. Dose-calibrator ‘maximum’ (CardioGen-82^®^) values were found to under-estimate the integrated ‘total’ (RUBY-FILL^®^) activity by ~ 24% at 50 mL·min^−1^. When these data were used to convert the CardioGen-82 values to a common measurement scale (integrated total activity) the CardioGen-82^®^ efficiency remained slightly lower than the RUBY-FILL^®^ system on average (88 ± 4% vs 95 ± 4%, *P* < 0.001). The efficiency of ^82^Rb production improved for both systems over the respective periods of clinical use.

**Conclusions:**

^82^Rb generator yield was significantly under-estimated using the CardioGen-82^®^ vs RUBY-FILL^®^ daily QA procedure. When generator yield was expressed as the integrated total activity for both systems, the estimated ^82^Rb production efficiency of the CardioGen-82^®^ system was ~ 7% lower than RUBY-FILL^®^ over the full period of clinical use.

**Electronic supplementary material:**

The online version of this article (10.1007/s12350-020-02200-6) contains supplementary material, which is available to authorized users.

## Introduction

Rubidium-82 (^82^Rb) is a widely used positron emission tomography (PET) tracer with a short half-life of ~ 75 seconds.^1^ Stress perfusion imaging with ^82^Rb is used widely in cardiac PET centers for the assessment of known or suspected coronary artery disease.^2^ The short half-life of ^82^Rb allows rapid sequential rest and stress imaging with low-radiation exposure to patients and medical staff.^3^,^4^ A mobile generator system is used to produce the ^82^Rb-chloride tracer on-demand from the parent radioisotope strontium-82 (^82^Sr) which has a half-life of ~ 25 days.^5^ The generator ion-exchange column is typically made of a hydrated tin-oxide, and loaded with 100 mCi (3.7 GBq) of ^82^Sr activity that decays continuously to produce ^82^Rb. The strong adsorption of ^82^Sr to tin-oxide compared to the weak binding of ^82^Rb enables the selective elution of ^82^Rb-chloride solution with standard physiologic saline.^6^ The ^82^Rb activity is then replenished quickly on the generator column (93.8-99.6% within 5-10 minutes, respectively) according to the properties of parent–daughter secular equilibrium.^7^

While the main components of ^82^Sr/^82^Rb generators are similar across different commercial systems, e.g. ion exchange column, saline supply and pump, generator and patient intravenous lines,^8^ the shelf-life (approved period for clinical use) can be different in clinical practice.^9^–^11^ The factors that influence generator shelf-life are the available daily ^82^Rb isotope yield and the total volume of saline eluted through the column, both of which must be within the approved limits for clinical perfusion imaging.^12^ Furthermore, the exact amount of delivered ^82^Rb activity and its infusion profile (e.g. constant flow-rate vs constant activity-rate) are important parameters for optimizing dynamic imaging accuracy and repeatability.^13^,^14^ In this context, the aims of the current study were to assess the isotope production efficiency (^82^Rb yield/^82^Sr parent activity) of two FDA-approved ^82^Sr/^82^Rb generators during their clinical shelf-life and to characterize the effect of different daily quality assurance (QA) procedures on measured ^82^Rb activity.

## Methods

### Quality Assurance Procedures

Sequential daily QA reports (N = 515) were reviewed from 9 CardioGen-82^®^ (Bracco Diagnostics Inc., Monroe, NJ) and 9 RUBY-FILL^®^ (Jubilant DraxImage, Kirkland, QC) generators used at Cardiac Imaging Nuclear Associates, Inc. (Los Angeles, CA) over a continuous period from January 2017 to May 2019. Parent ^82^Sr activity was calculated on a given date *Sr(d)* based on the radiopharmaceutical calibration certificate activity on the date of manufacturing *Sr(D*_*0*_*)* = 100 mCi (3.7 GBq), according to the known isotope decay, i.e.

1 $$ {\text{Sr}}\left( d \right) = {\text{Sr}}\left( {D_{0} } \right) \times e^{{ - \varLambda \left( {d - D_{0} } \right)}} $$

where $\varLambda = \ln \left( 2 \right)/25.35 \,{\text{days }}$ is the decay constant of ^82^Sr.^5^ The initial calibration activity of ^82^Sr parent was confirmed as 100 mCi (3.7 GBq) for all generators on the date of manufacturing (*D*_*0*_).

**Figure 1:**
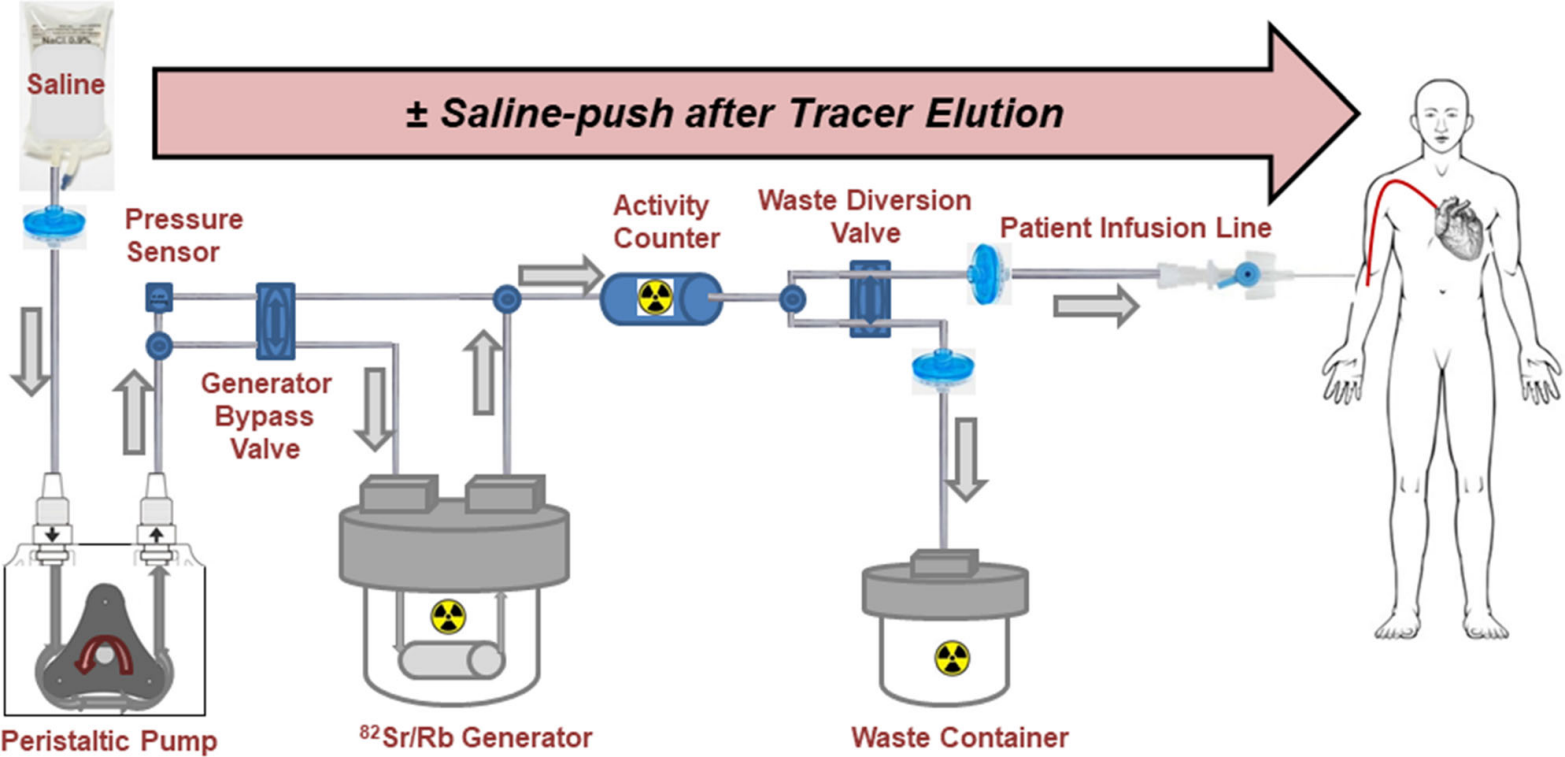
Components of the RUBY-FILL^®^^82^Rb generator and elution system. The CardioGen-82^®^ system is similar but uses a syringe pump instead of peristaltic pump and does not include the generator-bypass valve/tubing for saline-push

The operation of both ^82^Sr/^82^Rb elution systems follow similar principles as described before, and illustrated in Figure 1.^15^ Briefly, a syringe pump (CardioGen-82^®^) or peristaltic pump (RUBY-FILL^®^) is used to elute normal physiologic saline (0.9% NaCl USP) through the generator column. A system of tubing delivers the radioactive ^82^Rb eluate either to a sterile patient intravenous (IV) injection line or to a waste container. The CardioGen-82^®^ was designed for elution at a constant flow-rate of 50 mL·min^−1^. The RUBY-FILL^®^ elution system allows variable flow-rates from 15 to 30 mL·min^−1^. It has an additional generator-bypass line used to flush ^82^Rb activity out of the patient line at the end of elution.^16^ The bypass line can also be used with feedback control to standardize the shape of the infused activity profile, providing the same ‘square-wave’ injection regardless of the age of the generator.

**Figure 2:**
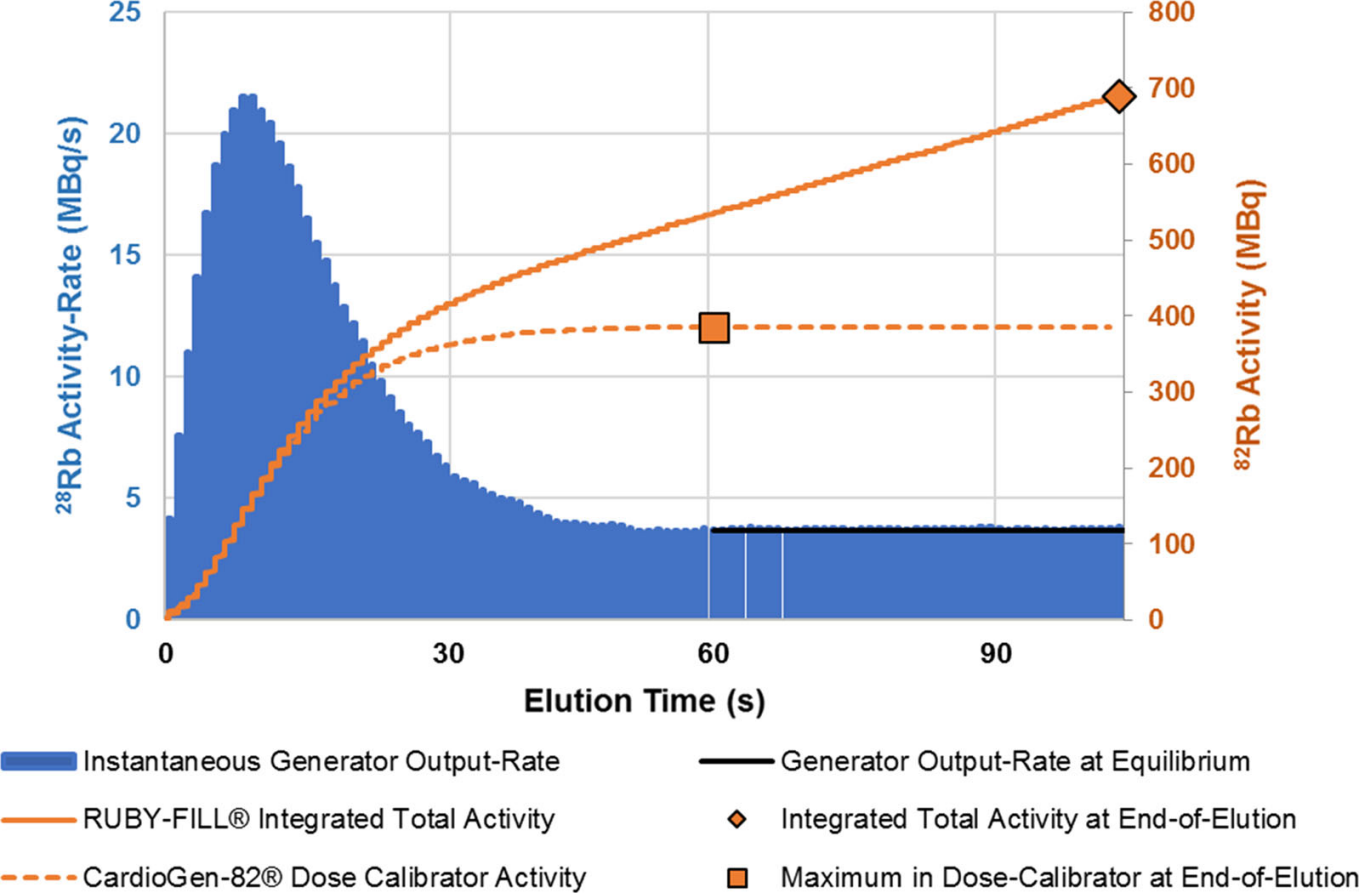
Example ^82^Rb generator activity output profiles from a RUBY-FILL^®^ test elution performed at 20 mL·min^−1^. ^82^Rb eluate activity (MBq/s, blue) as measured at the output of the patient infusion line over time. Total activity delivered to the patient or calibration vial (MBq, solid orange line) increases over-time according to the integrated area under the ^82^Rb eluate time–activity curve. Activity measured in a dose-calibrator vial (CardioGen-82^®^) increases initially over time, but then reaches a steady-state maximum value (MBq, dashed orange line) which is lower than the integrated total activity

The daily ^82^Rb calibration and breakthrough testing procedures use preset constant values for both the RUBY-FILL^®^ (35 mL at 20 mL·min^−1^) and CardioGen-82^®^ (50 mL at 50 mL·min^−1^) generators. The RUBY-FILL^®^ system reports the second-by-second integrated ‘total’ activity delivered during the daily calibration elution, as shown in Figure 2. The CardioGen-82^®^ system does not report the integrated total ^82^Rb activity as part of the daily QA procedures, but rather as the dose-calibrator ‘maximum’ value recorded at the end of the breakthrough testing elution.

For both generators, the ^82^Rb activity *A*_*F*_*(t)* is delivered to the outlet of the patient infusion line at an instantaneous output-rate (MBq/s) that varies as a function of elution time *t,* and elution flow-rate *F*. During daily calibration the RUBY-FILL^®^ system measures the delivered activity as the cumulative integrated ‘total’ of the ^82^Rb activity delivered over *T = 105* seconds duration of a 35 mL elution at *F* = 20 mL·min^−1^, i.e.

2 $$ {\text{Rb}}_{\text{TOTAL}} \left( T \right) = \mathop \smallint \limits_{0}^{T} A_{20} \left( t \right) {\text{d}}t_{ } $$

On the CardioGen-82^®^ system, the dose-calibrator maximum activity is measured in a collection vial at the end of the 50 mL elution at *F* = 50 mL·min^−1^, i.e. ${\text{Rb}}_{\text{MAX}} \left( {T = 60 \,{\text{seconds}}} \right)$ performed as part of the daily breakthrough testing (Supplemental Figure S1). Beyond the 60-seconds time-point, ^82^Rb activity in the vial reaches a maximum value representing a steady-state balance between rubidium activity output from the generator and isotope decay, according to:

3 $$ {\text{Rb}}_{\text{MAX}} \left( T \right) = \mathop \smallint \limits_{0}^{T} A_{50} \left( t \right) e^{{ - \lambda \left( {T - t} \right)}} {\text{d}}t_{ } $$

where $\lambda = \ln \left( 2 \right)/75.35\,{\text{seconds}}$ is the decay constant of ^82^Rb.^5^

The respective values, i.e. CardioGen-82^®^ ‘maximum’ and RUBY-FILL^®^ ‘total’ are reported here as the measured daily QA activity. The ^82^Rb yield is reported for both systems as the integrated total activity delivered over the time-course of an elution into the daily QA vial (Table 1).

**Table 1:** Activity, yield and efficiency methods for each elution system

Generator system	Daily QA reported ^82^Rb activity	Reported ^82^Rb yield	Calculated efficiency
RUBY-FILL^®^	Integrated total from calibration/breakthrough elution	Integrated total from calibration/breakthrough elution	Integrated total ^82^Rb yield/^82^Sr parent
CardioGen-82^®^	Dose Cal maximum from daily QA breakthrough elution	Integrated total estimated from daily QA and RUBY-FILL test elutions (1.3 × Max)	Integrated total ^82^Rb yield/^82^Sr parent

### CardioGen-82^®^ Correction Factor

On the RUBY-FILL^®^ system, the ^82^Rb yield is equal to the ‘Dose Delivered’ value shown on the daily QA calibration elution report (Supplemental Figure S2). To enable accurate comparison of ^82^Rb yield between the two systems, a correction factor was determined to scale the CardioGen-82^®^ daily QA activity (dose-calibrator maximum values) to the integrated total values that would be expected using the RUBY-FILL^®^ calibration procedure.

The CardioGen-82^®^ system operates at a fixed elution flow-rate of 50 mL·min^−1^, whereas RUBY-FILL^®^ can operate over a range of flow-rates from 15 to 30 mL·min^−1^. Since there was no single elution flow-rate that could be used to compare both systems directly, a series of test elutions (60-seconds duration) was performed using the RUBY-FILL^®^ generator (n = 6 days each) to measure the dose-calibrator ‘maximum’ activities produced over the range of available flow-rates (15, 20, 25 and 30 mL·min^−1^). These values were expressed relative to the true integrated total activity measured on each day and plotted as a function of the elution flow-rate. Because the changes with flow-rate did not appear to be linear, two non-linear regression (logarithmic and power function) models were used to fit these data and extrapolate the maximum/total activity ratio expected at 50 mL·min^−1^. The inverse ratio was then used as a correction factor to scale the measured CardioGen-82^®^ dose-calibrator maximum values to the equivalent integrated total activity values (^82^Rb yield), as measured by the RUBY-FILL^®^ system.

### Generator Performance

For the RUBY-FILL^®^ test elutions, percent error in the integrated total activity (actual/requested – 1) × 100% was determined as a function of the elution flow-rate. The actual elution flow-rates were also verified by dividing the volume of the eluate (mL) by the elution time (minutes) and plotting the elution flow-rate error (actual/requested – 1) × 100% as a function of the requested flow-rate. For both systems, the generator efficiency was reported as the ratio of ^82^Rb yield (integrated total activity) divided by the ^82^Sr parent activity available over the course of the generator shelf-life:

4 $$ {\text{Efficiency}} = {}_{ }^{82} {\text{Rb}}\;{\text{yield}} \div {}_{ }^{82} {\text{Sr}}\;{\text{parent}}\;{\text{activity}} \times 100\% $$

### Statistical Analysis

All values are presented as mean ± standard deviation. Correlations of continuous data were assessed using parametric bivariate analysis. Logarithmic and power function regression models were used for the calibration data extrapolation. Comparison of data between generators was performed with a two-way analysis of variance (ANOVA) assuming unequal variances, and statistical significance was considered using *P* < 0.05. Statistical testing was performed using Excel^®^ v2017 (Microsoft Corp, Redmond, WA).

## Results

### Daily QA Measurements

**Figure 3:**
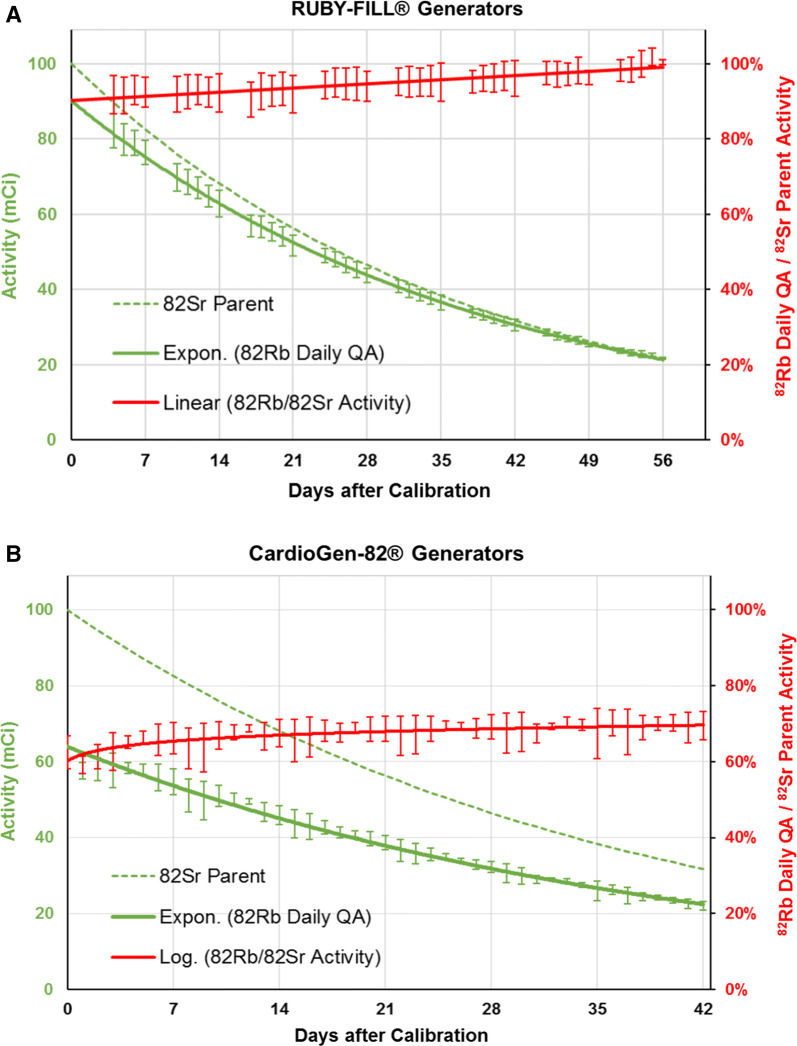
^82^Rb daily QA activity (solid green lines) measured on RUBY-FILL^®^ (**A**) and CardioGen-82^®^ (**B**) generator systems. Activity is shown in mCi (GBq ÷ 37) on the left axis, and as a percent of the ^82^Sr parent activity available on each day of use (solid red lines) on the right axis

^82^Rb QA activity values were measured for 240 days with the CardioGen-82^®^ system and for 275 days with RUBY-FILL^®^ (N = 515 total). The average duration of clinical use was 32% longer with the RUBY-FILL^®^ vs CardioGen-82^®^ systems (54 ± 1 vs 41 ± 2 days, *P* < 0.001) as expected given the different generator expiration dates (60 vs 42 days) and elution volume limits (30 vs 17 L) listed in the respective package inserts.^9^,^11^ The measured daily QA activity values are shown as a function of generator age (day of use) for both systems in Figure 3. On the first day of clinical use, the QA activity measured on the RUBY-FILL^®^ system was 32% higher vs CardioGen-82^®^ (Table 2), however on the last day of use the activity was the same for both systems (~ 800 MBq [22 mCi] on day 56 for RUBY-FILL^®^ or day 42 for CardioGen-82^®^). When the ^82^Rb QA activity was expressed as a percentage of the ^82^Sr parent activity available on a given day, the average value for the CardioGen-82^®^ generator was 67.3 ± 3.4% compared to 95.0 ± 3.8% for RUBY-FILL^®^ (*P* < 0.001) over the full period of clinical use.

**Table 2:** ^82^Rb generator daily QA activity (n = 9 each)

Generator system	Integral (yield)		Maximum (steady-state)	
	GBq (mCi) on first day of use	GBq (mCi) on last day of use	GBq (mCi) on first day of use	GBq (mCi) on last day of use
RUBY-FILL^®^*	3.04 ± 0.17 (82.2 ± 4.6) on day 4	0.80 ± 0.01 (21.7 ± 0.1) on day 56	2.31 ± 0.13 (62.6 ± 3.5) on day 4	0.61 ± 0.01 (16.5 ± 0.08) on day 56
CardioGen-82^®^**	3.03 ± 0.21 (82.0 ± 5.6) on day 0	1.07 ± 0.06 (28.9 ± 1.6) on day 42	2.31 ± 0.16 (62.4 ± 4.3) on day 0	0.81 ± 0.04 (22.0 ± 1.2) on day 42

Values are mean ± standard deviation. mCi values = GBq × 27 mCi/GBq

* RUBY-FILL Maximum values = Integral values ÷ 1.314 correction-factor

**CardioGen-82 Integral values = Maximum values × 1.314 correction-factor

### CardioGen-82^®^ Correction Factor

The difference in daily QA activities was due, in part, to the different measurement techniques (dose-calibrator maximum vs integrated total activity), therefore the corresponding values for the CardioGen-82^®^ system were derived using the result of the RUBY-FILL^®^ test elutions. Typical ^82^Rb time activity curves (TACs) for the test elutions performed at different flow-rates are shown in Supplemental Figure S3. As expected, the 20-mL·min^−1^ test elution and calibration curves were nearly identical during the common 60-second elution interval. Percent error in the actual delivered vs. requested activity was consistently below 0.2% over the full range of flow-rates (Supplemental Figure S4A) confirming accuracy of the RUBY-FILL^®^ elution system over the full range of flow-rates.^6^ Error in the actual vs requested flow-rate was below 6% for all elutions (Supplemental Figure S4B) demonstrating accuracy of the RUBY-FILL^®^ peristaltic pump over the range of elution flow-rates tested. There was a negative correlation of flow-rate error with increasing flow-rate (*R*^2^ = 0.85), without significant error at 20-25 mL·min^−1^.

**Figure 4:**
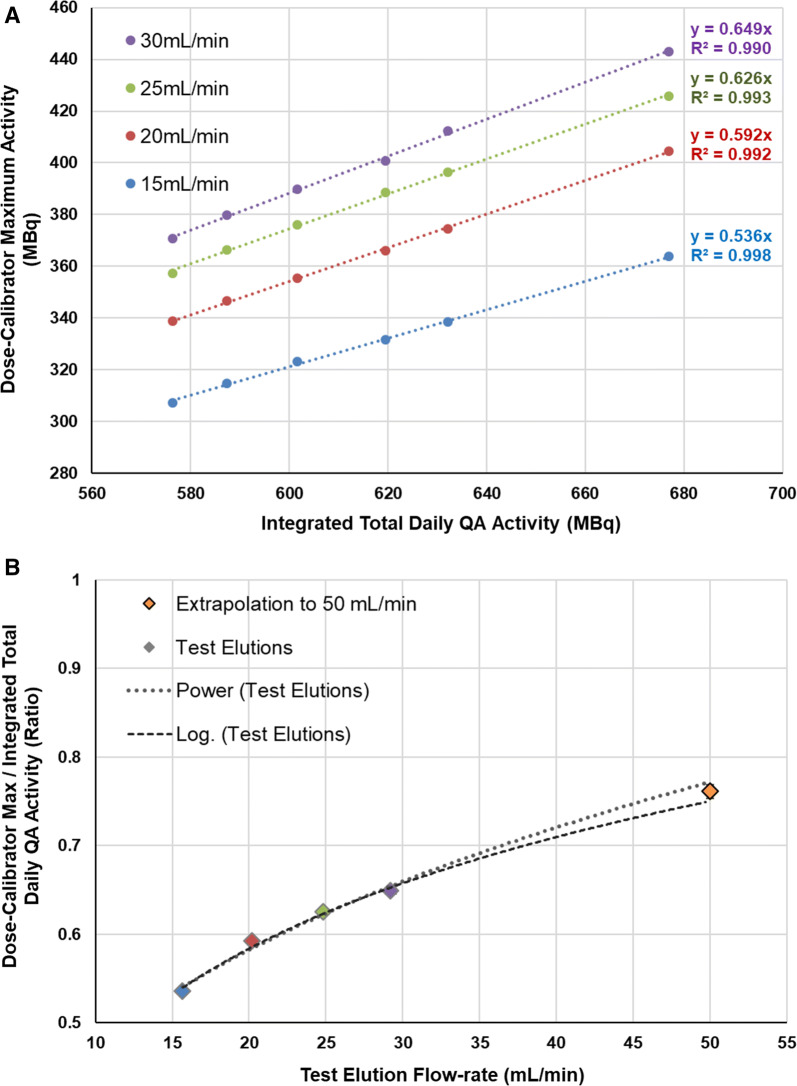
RUBY-FILL^®^ test elution results (n = 6 each). At each elution flow-rate, the dose-calibrator maximum activity (**A**) is a constant fraction (slope) of the integrated total QA activity. The corresponding ratios of Dose-calibrator Maximum/Integrated Total calibration activity (**B**) are extrapolated to the value of 0.761 that would be expected using a 50 mL @50 mL·min^−1^ elution. Standard deviation error bars (~ 1%) are smaller than the plotted symbol size

At each flow-rate tested, the dose-calibrator maximum activity was very highly correlated with the integrated total (*R*^2^ > 0.99) as shown in Figure 4A. The maximum/total activity ratios (slope of the lines in Figure 4A) increased significantly as a function of flow-rate, as summarized in Table 3. At an extrapolated flow-rate of 50 mL·min^−1^, the dose-calibrator maximum/total activity ratio was estimated as 0.750 ± 0.007 and 0.772 ± 0.008 using the logarithmic and power function models, respectively (Figure 4B). Both the non-linear functions had higher *R*^2^ (> 0.98) than a linear model fit to the data (*R*^2^ = 0.96). The average value (0.761 ± 0.008) was used to compute the CardioGen-82^®^ correction-factor as 1/0.761 = 1.314 ± 0.013.

**Table 3:** RUBY-FILL^®^ test elution activity ratios (n = 6 each)

Elution rate	Elution maximum ÷ calibration total (from Figure 4A)	Elution maximum ÷ elution total (from Figure S3A)
15 mL·min^−1^	0.536 ± 0.002	0.830 ± 0.005
20 mL·min^−1^	0.592 ± 0.003	0.775 ± 0.005
25 mL·min^−1^	0.626 ± 0.003	0.724 ± 0.007
30 mL·min^−1^	0.649 ± 0.004	0.684 ± 0.007
50 mL·min^−1^	0.761 ± 0.008 *	0.51 ± 0.07 *

*extrapolated using mean  ±  SD of logarithmic and power function models

### Generator Yield and Efficiency

When the CardioGen-82^®^ dose-calibrator maximum ^82^Rb values were multiplied by the estimated correction-factor (1.314), the integrated total ^82^Rb yield on the first day of use (day 0) was 3.03 ± 0.21 GBq, very similar to the value of 3.04 ± 0.17 GBq measured on the RUBY-FILL^®^ system (day 4) as shown in Table 2.

**Figure 5:**
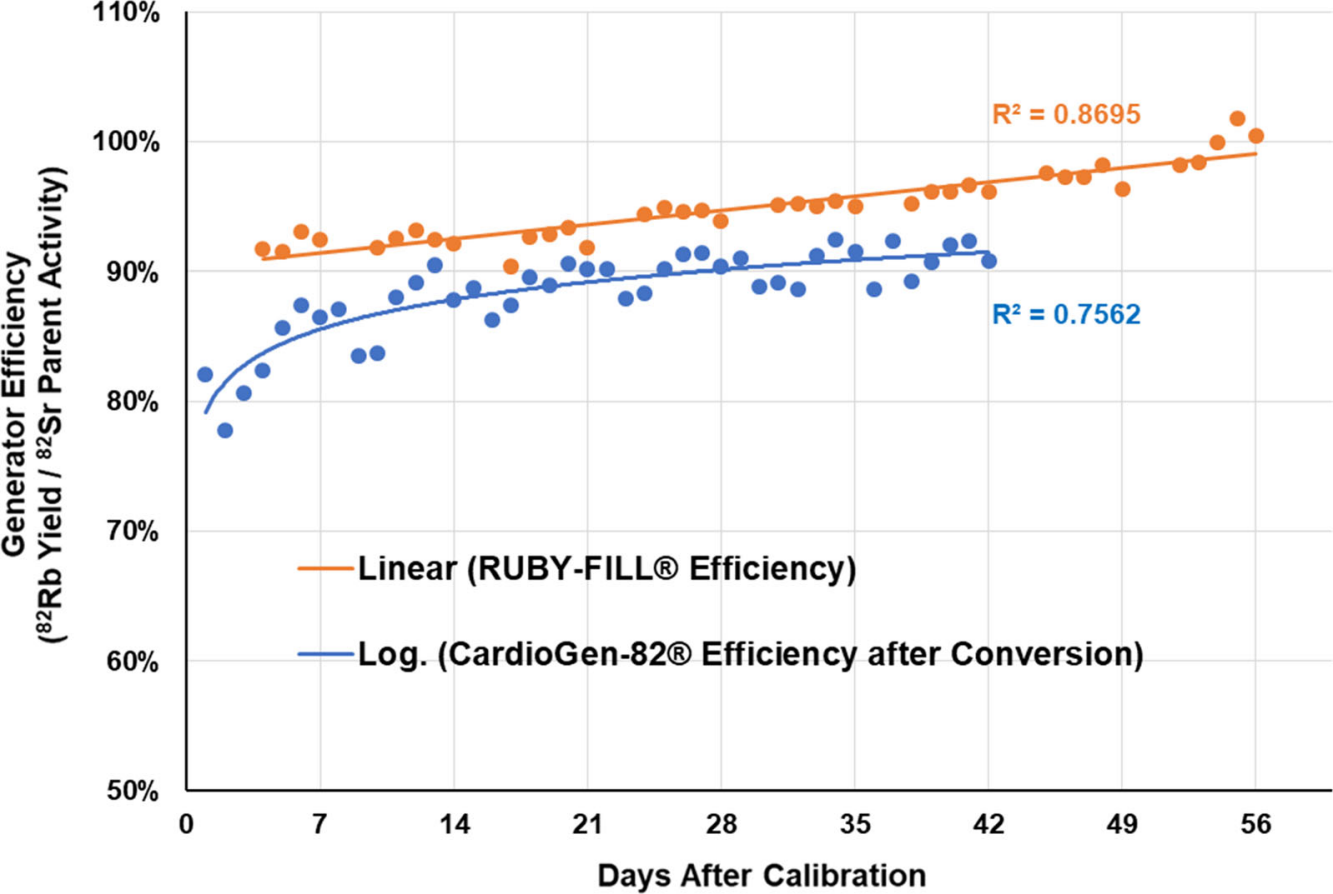
^82^Rb isotope production efficiency of the RUBY-FILL^®^ and CardioGen-82^®^ systems over the clinical shelf-life of N = 9 generators each. ^82^Rb yield values were measured for RUBY-FILL^®^ (and estimated for CardioGen-82^®^) using 35 mL elution at 20 mL·min^−1^

The corresponding CardioGen-82^®^ efficiency was estimated as 88.4 ± 4.4% (Figure 5) which was slightly lower than the RUBY-FILL^®^ efficiency of 95.0 ± 3.8% measured over the full period of clinical use (*P* < 0.001).

## Discussion

^82^Rb stress PET has become a routine method for myocardial perfusion imaging including the quantification of absolute myocardial blood flow (mL·min^−1^·g^−1^). The utility of ^82^Rb PET to diagnose coronary artery disease and to guide appropriate treatment for CAD patients has been well-established previously.^17^ The documented prognostic value of ^82^Rb PET perfusion scanning,^2^,^18^,^19^ its low-radiation exposure to patients and medical staff,^1^ and the availability of approved on-site ^82^Sr/^82^Rb generators^20^ plays a key role in the increasing use of this modality. The number of imaging centers using ^82^Rb PET has risen dramatically since the initial commercial approval (CardioGen-82^®^) and subsequent addition (RUBY-FILL^®^) of these ^82^Rb generator systems.^21^ With this increase in clinical capacity and diversity of generator systems, the need for standardized patient dosing is critical, as the net amount of injected activity ultimately determines the PET image quality and absorbed radiation dose to the patient.^22^

This study compared the ^82^Rb production efficiency of the two-generator systems available for cardiac PET perfusion imaging, and characterized the daily QA procedure effects on measured isotope yield. The main findings indicated: (1) ^82^Rb yield of the CardioGen-82^®^ generator was under-estimated by ~ 24% using dose-calibrator maximum values for daily QA; (2) a correction-factor of ~ 1.3 may be used to convert CardioGen-82^®^ daily QA values to equivalent integrated total ^82^Rb activity as measured by the RUBY-FILL^®^ system; (3) ^82^Rb isotope yield and production efficiency were estimated to be ~ 7% higher for the RUBY-FILL^®^ vs CardioGen-82^®^ system, as measured over a clinical shelf-life that was ~ 30% longer.

A novel method was developed to bring the ^82^Rb isotope yield measured by two different generators to a common scale for accurate comparison. The CardioGen-82^®^ elution protocol recommends the patient maximum volume to be set at 50 mL and the patient dose at 1480 MBq (40 mCi), with a range of 1110-2220 MBq (30-60 mCi).^11^,^23^ The RUBY-FILL^®^ system is user-adjustable for dose (e.g. 10-30 MBq·kg^−1^ [0.27-0.81 mCi·kg^−1^] body weight) and flow-rate (15-30 mL·min^−1^) or infusion time (10-120 seconds).^9^,^24^ The infused patient activity is also reported differently by the two systems. RUBY-FILL^®^ reports only the integrated total activity for both daily QA and patient studies. The CardioGen-82^®^ system reports the integrated ‘Patient Dose’ and the ‘End-of-Infusion’ activity values, which must be calibrated at the same activity used for patient elutions. Neither of these activity values appear to be accurate on the daily QA breakthrough elution reports, therefore, they should not be used as a measure of CardioGen-82^®^^82^Rb isotope yield.

Both generator systems showed a small increase in ^82^Rb isotope production efficiency over time (Figure 5). The mechanism for this observed improvement is not completely understood but may be due to migration of ^82^Sr activity away from the column inlet, where it can be eluted from the column more completely. The difference in measured efficiency between vendors may be due to alternative chemical preparation/conditioning of the column resin and/or physical geometry/volume of exchanger in the generator columns, but these are proprietary data and difficult to confirm.

Three-dimensional (3D) PET systems are the current commercial standard which provides higher sensitivity for data acquisition, compared to previous generation 2D scanners.^20^ However, highly sensitive signal acquisition carries a higher risk for detector saturation which can result in the over-estimation of myocardial blood flow. The saturation effect on dynamic imaging can be prevented by adjusting the injected activity for body weight to optimize the amount of tracer in the bolus first-pass transit.^13^ To optimize the injected ^82^Rb activity in regard to the camera sensitivity, the difference between requested vs injected activity (and volume), maximum vs integral activity, and constant vs variable infusion flow rate are important parameters that need to be taken into consideration. In this context, the current study provides insights to the technical aspects of ^82^Sr/^82^Rb generators that can affect the image quality and myocardial blood flow quantification.^14^ Our results indicated that the RUBY-FILL^®^ elution activity and flow errors (in proportion to the requested values) are exceedingly small, which confirms the high precision of this infusion system. Moody et al. have shown previously that the error in actual measured/requested dose for the CardioGen-82^®^ generator was up to 50% for a requested activity of 370 MBq (10 mCi) and 33% for a requested activity of 550 MBq (15 mCi), and decreased as the requested activity approached 30 mCi (1110 MBq).^25^ In our test elutions with RUBY-FILL^®^, the range of requested elution activity was ~ 400 to 600 MBq (11 to 16 mCi), and the elution activity error was negligible (less than 0.2%) due to the accurate modeling of eluate transport delay and isotope decay en-route from the generator to the outlet of the patient infusion line.^6^

Secondary analysis of the integrated total activity from the RUBY-FILL^®^ test elutions (Supplemental Figure S5) demonstrated similar trends compared to the primary analysis of the dose-calibrator maximum values in Figure 4. The integrated total activity increased consistently as a function of elution flow-rate, up to a peak value 95% of the daily QA calibration activity, using 30 mL at 30 mL·min^−1^ elution. These data also suggest that if the RUBY-FILL^®^ generator was eluted using the CardioGen-82^®^ protocol (50 mL at 50 mL·min^−1^), then the ^82^Rb yield could increase to ~ 120% of the daily QA calibration activity.

Clinical procedure guidelines published by the Society of Nuclear Medicine^26^ recommend that radiopharmaceutical doses should be dispensed and subsequently administered to patients within 10% and 20% of those indicated by the prescribing physician. The U.S. NRC Regulations (10 CFR 35.63) on the use of medical isotopes state “… a licensee may not use a dosage if the dosage does not fall within the prescribed dosage range or if the dosage differs from the prescribed dosage by more than 20 percent”. 27 The most recent EANM guidelines for PET tumor imaging indicate that for automated administration “actual administered activity may not deviate by more than 3% from that indicated by the device”.^28^ The activity error data from the RUBY-FILL^®^ generator (Figure S4A) show that this system can meet even these most strict criteria, with actual vs requested activity errors of < 0.11% over all test elutions performed. In comparison, the printed ‘End-of-Infusion’ activity values from the CardioGen-82^®^ breakthrough elution reports appeared to under-estimate the measured dose-calibrator maximum values by ~ 30%, even when using the recommended elution settings of 50 mL at 50 mL·min^−1^. This underestimation may be due, in part, to the fact that this generator is typically calibrated using a requested ^82^Rb activity which is less than the total yield available on a given day, and subsequent elutions are only recommended using the same calibration activity.

The accuracy of ^82^Rb PET dosing is of clinical significance when the imaging results of different PET centers are compared or pooled together as part of multicenter imaging trials.^24^ For example, these results may have important implications for the calculation of patient absorbed radiation doses. Effective dose (ED) estimates have been published recently for ^82^Rb PET in the range of 0.80 and 1.1 mSv·GBq^−1^ based on PET studies using the RUBY-FILL^®^ and CardioGen-82^®^ generators, respectively.^3^,^4^ These radiation ED values are calculated using PET image-derived activity values divided (normalized) by the total injected activity, as reported by the respective generator systems. Because of this inverse relationship, *higher* ED values would be expected from PET studies performed using a generator system which reports *lower* administered ^82^Rb activity. In fact, the ratio of ED estimates from the RUBY-FILL^®^/CardioGen-82^®^ dosimetry papers (0.73) is similar to the CardioGen-82^®^ under-estimation of activity reported in the present study (0.76), providing a possible explanation for the discrepancy between these independently reported ED values. Our results suggest that the lower ED values reported by Hunter et al. using PET studies performed with the RUBY-FILL^®^ system may be more accurate, because the same integrated total activity method of measurement is used both during daily QA calibration and during patient elutions.^3^

### Limitations

The CardioGen-82^®^ correction-factor was calculated using the RUBY-FILL^®^ daily QA protocol (integrated total activity of 35 mL elution @ 20 mL·min^−1^) as the reference standard to enable accurate comparison of ^82^Rb isotope yield and production efficiency between the two systems. The results of this study do not suggest that all generator systems should use the same RUBY-FILL^®^ elution settings above for daily QA, but rather that the dose-calibrator maximum or ‘steady-state’ value measured in a calibration vial consistently under-estimates the integrated total activity delivered to the patient (Figure 2), and that this under-estimation is a function of the elution volume and flow-rate (Figure 4B). The integrated total activity could have been referenced instead to the CardioGen-82^®^ calibration elution settings (50 mL @ 50 mL·min^−1^) in which case the RUBY-FILL^®^ calibration values would have been multiplied by the extrapolated value of 0.761 for inter-comparison, as shown in Table 2.

Direct comparison of the activity profiles using the same elution flow-rate on both systems was not feasible in this study. The RUBY-FILL^®^ maximum flow-rate is 30 mL·min^−1^, whereas CardioGen-82^®^ uses a constant 50 mL·min^−1^ elution flow-rate. The CardioGen-82^®^ system does include a switch to use lower flow-rates (20 or 35 mL·min^−1^), but these are not part of the approved clinical labeling.^11^ Therefore, direct comparison could be performed in future studies if CardioGen-82^®^ daily QA was also measured at a flow-rate of 20 mL·min^−1^. In the current study, extrapolation of the RUBY-FILL^®^ test elution data from 30 to 50 mL·min^−1^ was associated with a certain margin of error in estimation of the CardioGen-82^®^ correction-factor. Although the logarithmic and power functions provided excellent fits to these data (*R*^2^ > 0.98), the extrapolated values should be interpreted with a degree of caution.

## Conclusions

While both ^82^Rb generators use a similar physical design to produce and administer the radiotracer, differences in the daily QA methods were shown to impact the measured ^82^Rb yield and efficiency. These characteristics of ^82^Sr/^82^Rb generators are of critical importance for accurate patient dosing, in particular when the myocardial perfusion imaging results from different cardiac PET imaging centers are compared or combined for multicenter clinical trials.

## New Knowledge Gained

The RUBY-FILL^®^ generator appears to have slightly higher ^82^Rb isotope production efficiency compared to CardioGen-82^®^, as well as a longer shelf-life and small elution activity error.

## Electronic supplementary material

Below is the link to the electronic supplementary material.

Electronic supplementary material 1 (DOCX 902 kb)

Electronic supplementary material 2 (PPTX 474 kb)

## Abbreviations

ED: Effective dose
PET: Positron emission tomography
QA: Quality assurance
^82^Rb: Rubidium-82
^82^Sr: Strontium-82
