# Supplementary material for: Rubidium-82 generator yield and efficiency for PET perfusion imaging: Comparison of two clinical systems
Source: J Nucl Cardiol. 2020 May 20;27(5):1728–38. doi: 10.1007/s12350-020-02200-6 (PMC7599151; doi:10.1007/s12350-020-02200-6)
Supplement: Supplementary file 1 — Electronic supplementary material 1 (DOCX 902 kb) [file 12350_2020_2200_MOESM1_ESM.docx]

**Supplemental Figure S1**. CardioGen-82 Daily QA Breakthrough Worksheet (top) and example Patient Elution Report (bottom left and right). Daily QA activity was measured manually using the dose-calibrator maximum ^82^Rb reading at the end of the breakthrough testing elution, and the decay-corrected reading was recorded on the Worksheet as shown in the red box.

**Supplemental Figure S2**. RUBY-FILL Daily QA Calibration and Breakthrough elution report. The ‘Dose Delivered’ is the integrated total Daily QA activity (shown in green) as measured during a 35 mL elution at 20 mL/min. The measured dose calibrator values are shown in blue, which reach a steady-state maximum value typically 60-90 seconds after the start of elution.


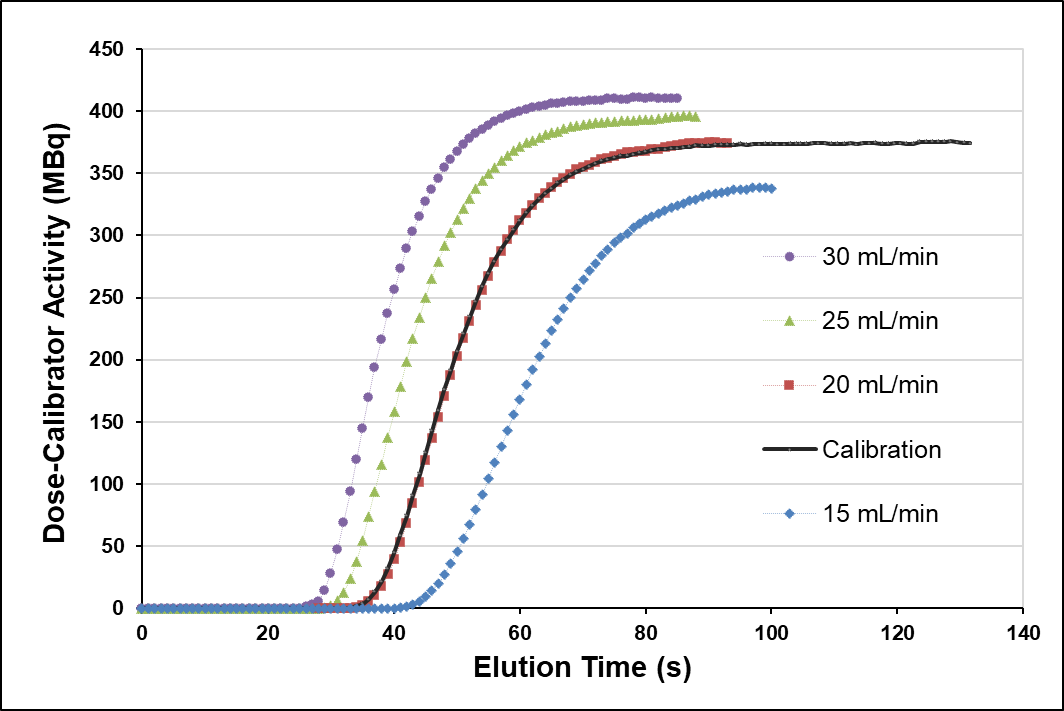


**Supplemental Figure S3**. RUBY-FILL® test elutions (60 seconds each) measured on day 3 out of 6. The dose-calibrator maximum activity increases as a function of elution flow-rate, because there is less time for ^82^Rb decay during transit from the generator to the dose-calibrator.

| **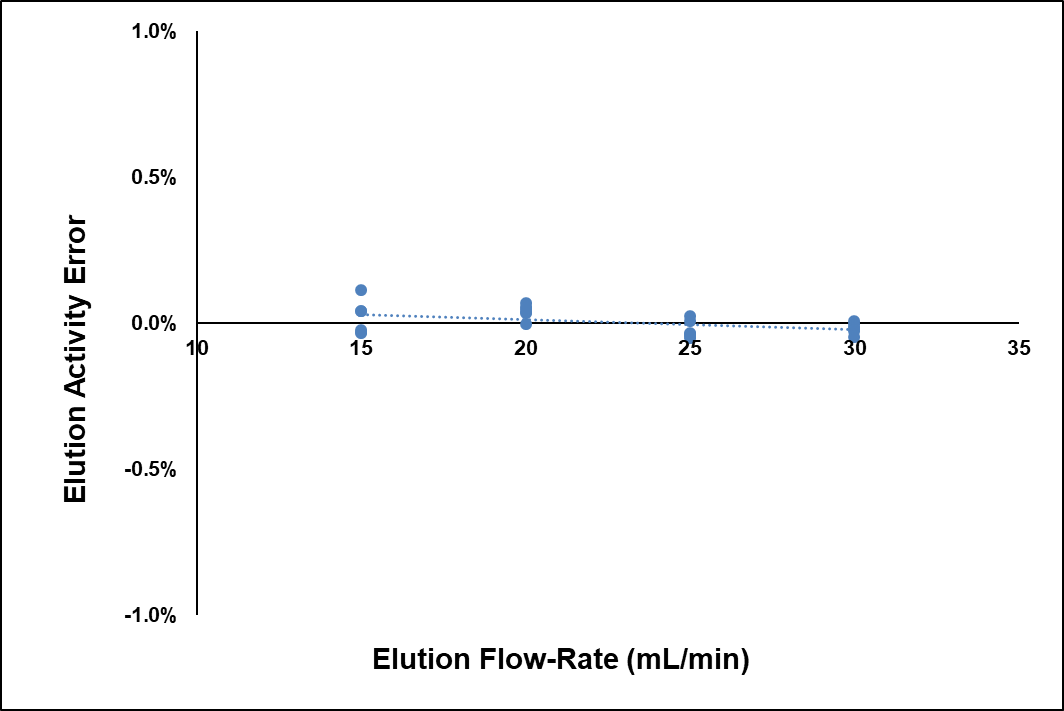A.** |
| --- |
| **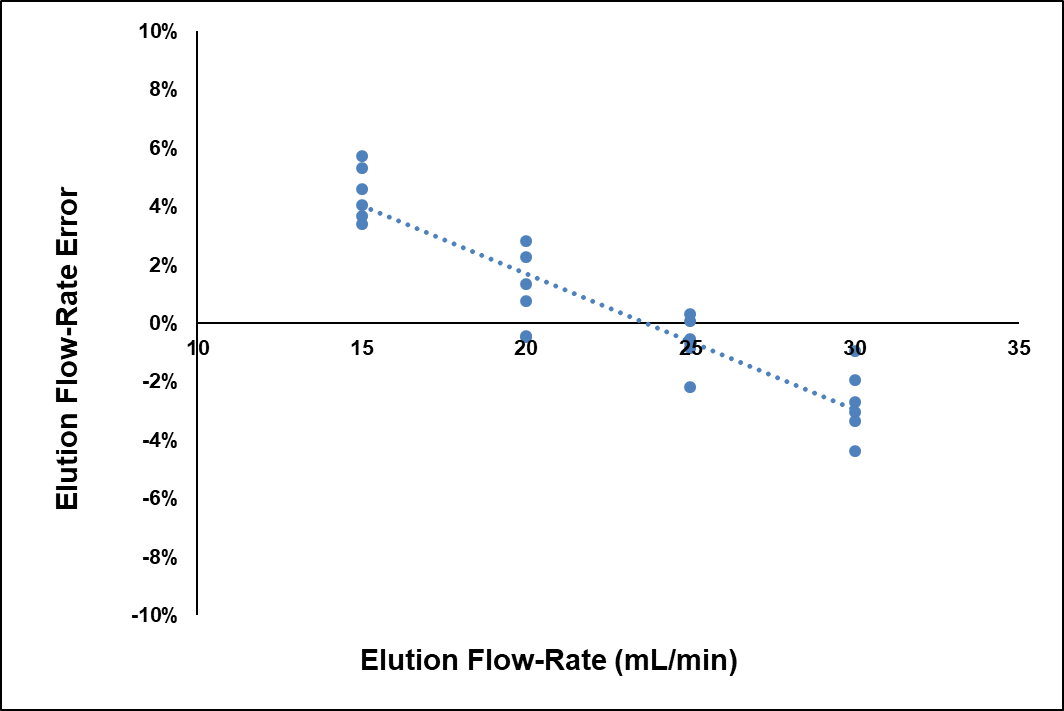B.** |

**Supplemental Figure S4**. RUBY-FILL® test elution results (n=6 days). The error in delivered vs requested activity (A) was less than 0.1% over all elution flow-rates. The measured elution flow-rate error (B) decreased with increasing flow-rate.

| \| **A. 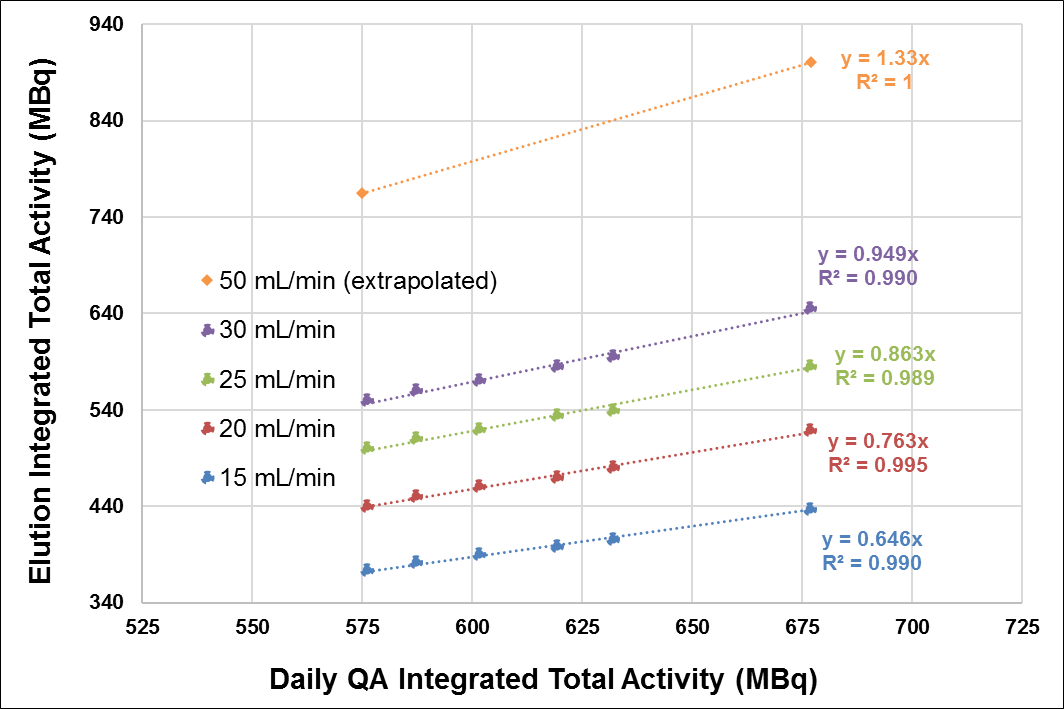**  **B. 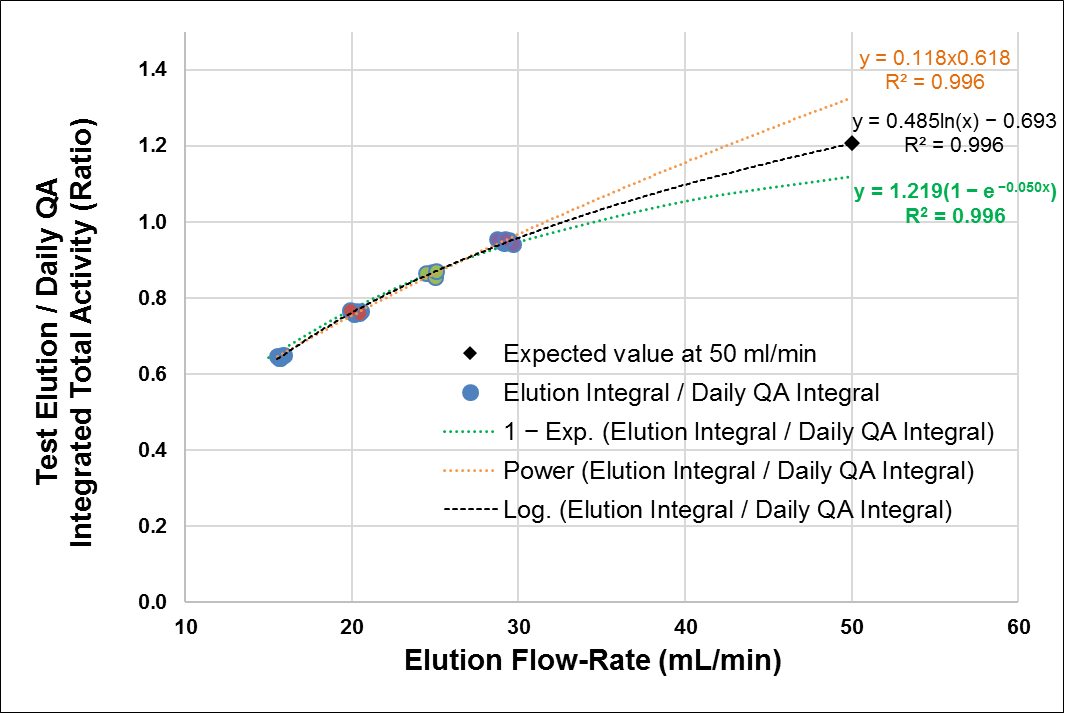** \| \| --- \| \|  \| |
| --- | --- | --- |

**Supplemental Figure S5**. RUBY-FILL® test elution results (n=6). At each flow-rate, the integrated total activity of the test elution (A) is a constant fraction (slope) of the daily QA integrated total activity. The corresponding Test Elution / Daily QA Integrated Total activity ratios (B) are extrapolated to the value of ~1.2 that would be expected using 50 mL @50 mL/min elution.

**
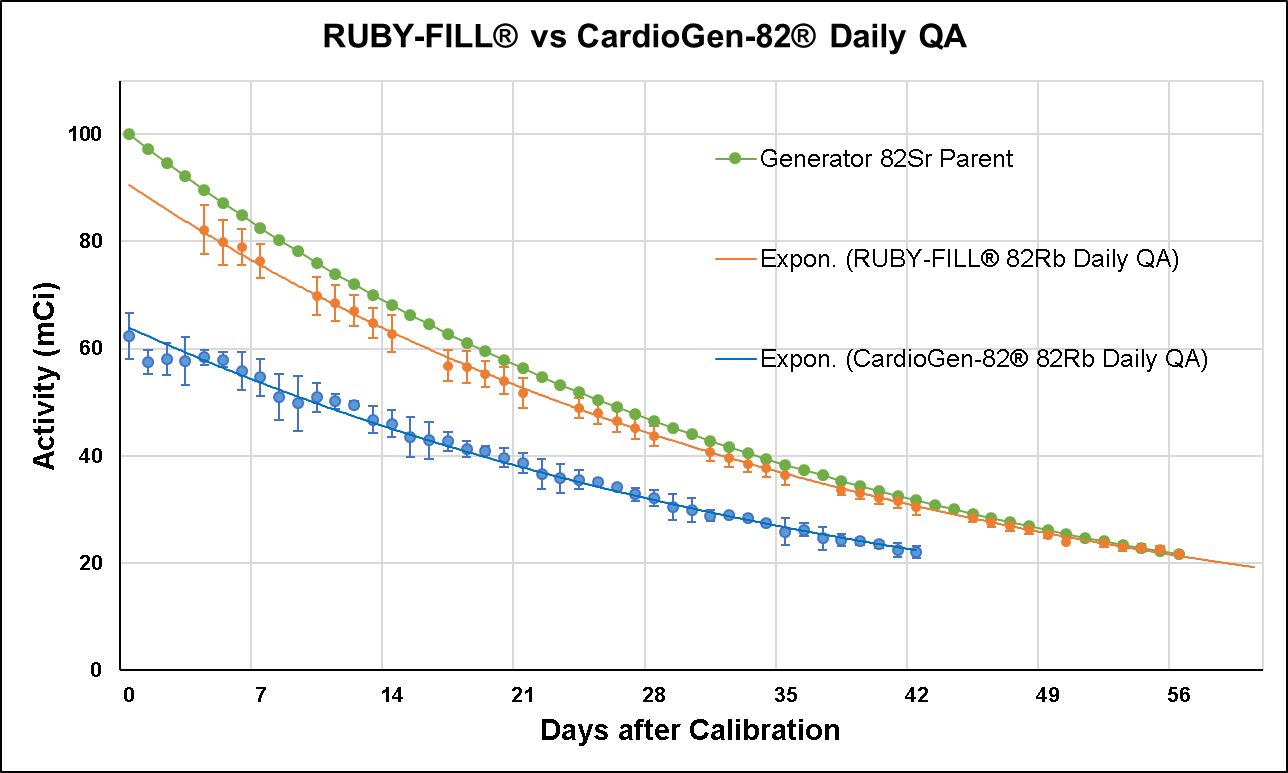
A.**

**
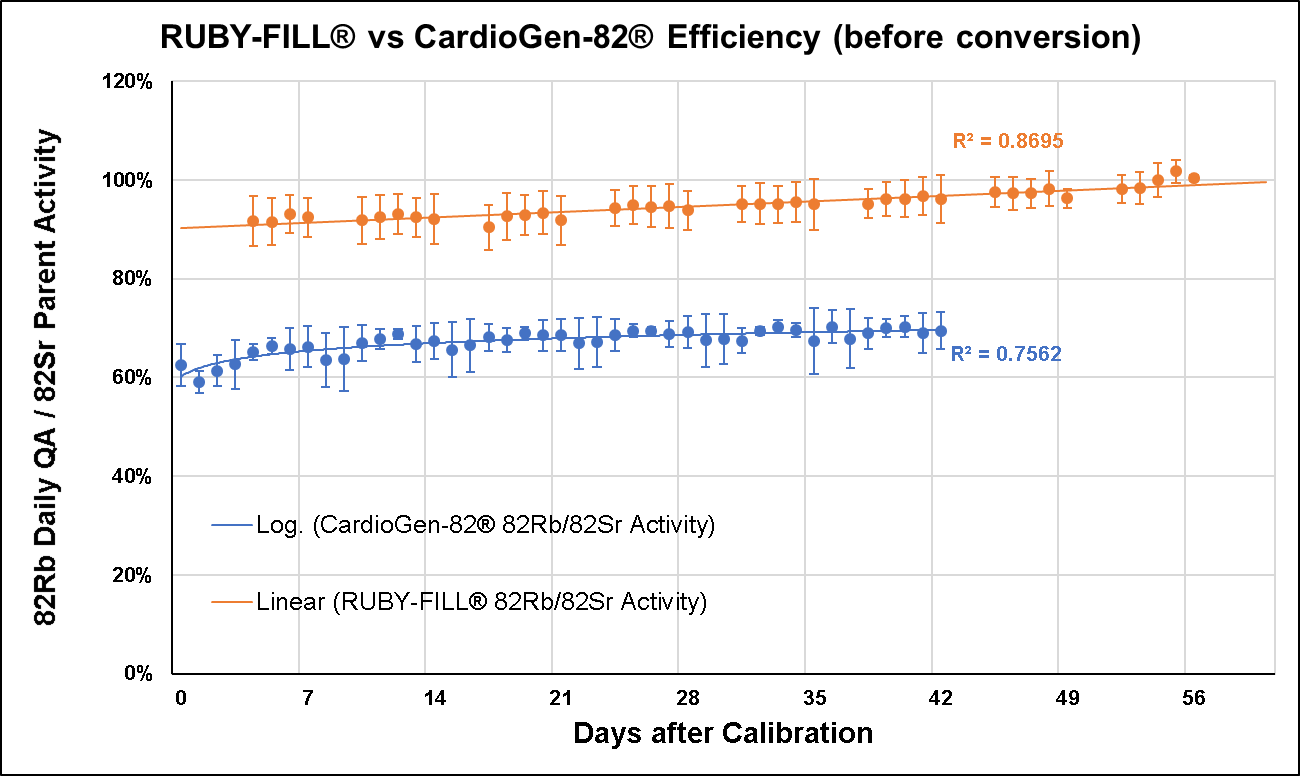
B.**

**Supplemental Figure S6**. ^82^Rb isotope production efficiency of the RUBY-FILL® (95 ± 4%) and CardioGen-82® (67 ± 3%) systems over the clinical shelf-life (N=9 generators each) before conversion to the same scale.
