# Supplementary material for: Rubidium-82 generator yield and efficiency for PET perfusion imaging: Comparison of two clinical systems
Source: J Nucl Cardiol. 2020 May 20;27(5):1728–38. doi: 10.1007/s12350-020-02200-6 (PMC7599151; doi:10.1007/s12350-020-02200-6)
Supplement: Supplementary file 2 — Electronic supplementary material 2 (PPTX 474 kb) [file 12350_2020_2200_MOESM2_ESM.pptx]

## Slide 1
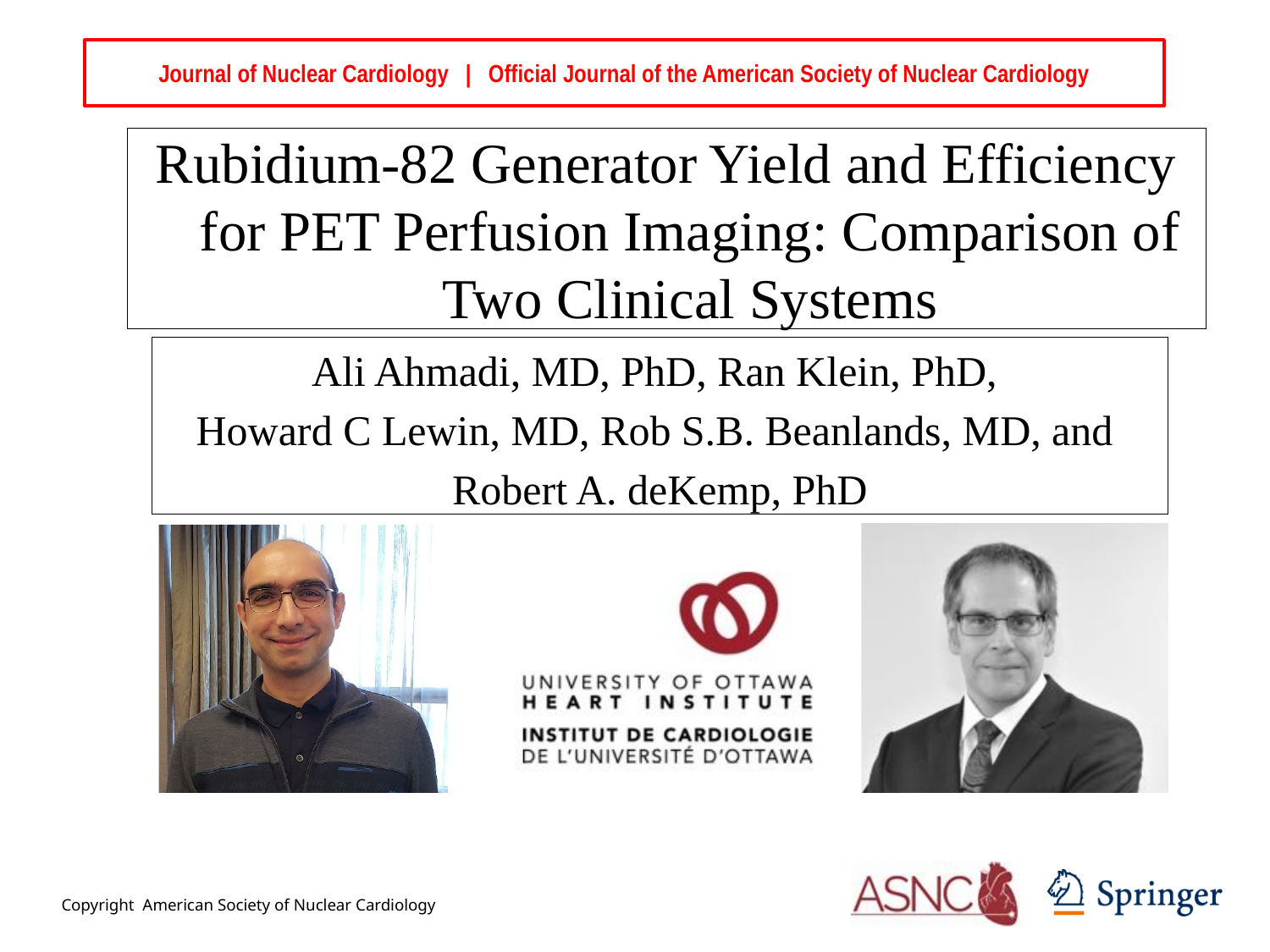

Journal of Nuclear Cardiology | Official Journal of the American Society of Nuclear Cardiology
# Rubidium-82 Generator Yield and Efficiency for PET Perfusion Imaging: Comparison of Two Clinical Systems
Ali Ahmadi, MD, PhD, Ran Klein, PhD,
Howard C Lewin, MD, Rob S.B. Beanlands, MD, and
Robert A. deKemp, PhD
Head shot of author
required
Copyright American Society of Nuclear Cardiology

## Slide 2
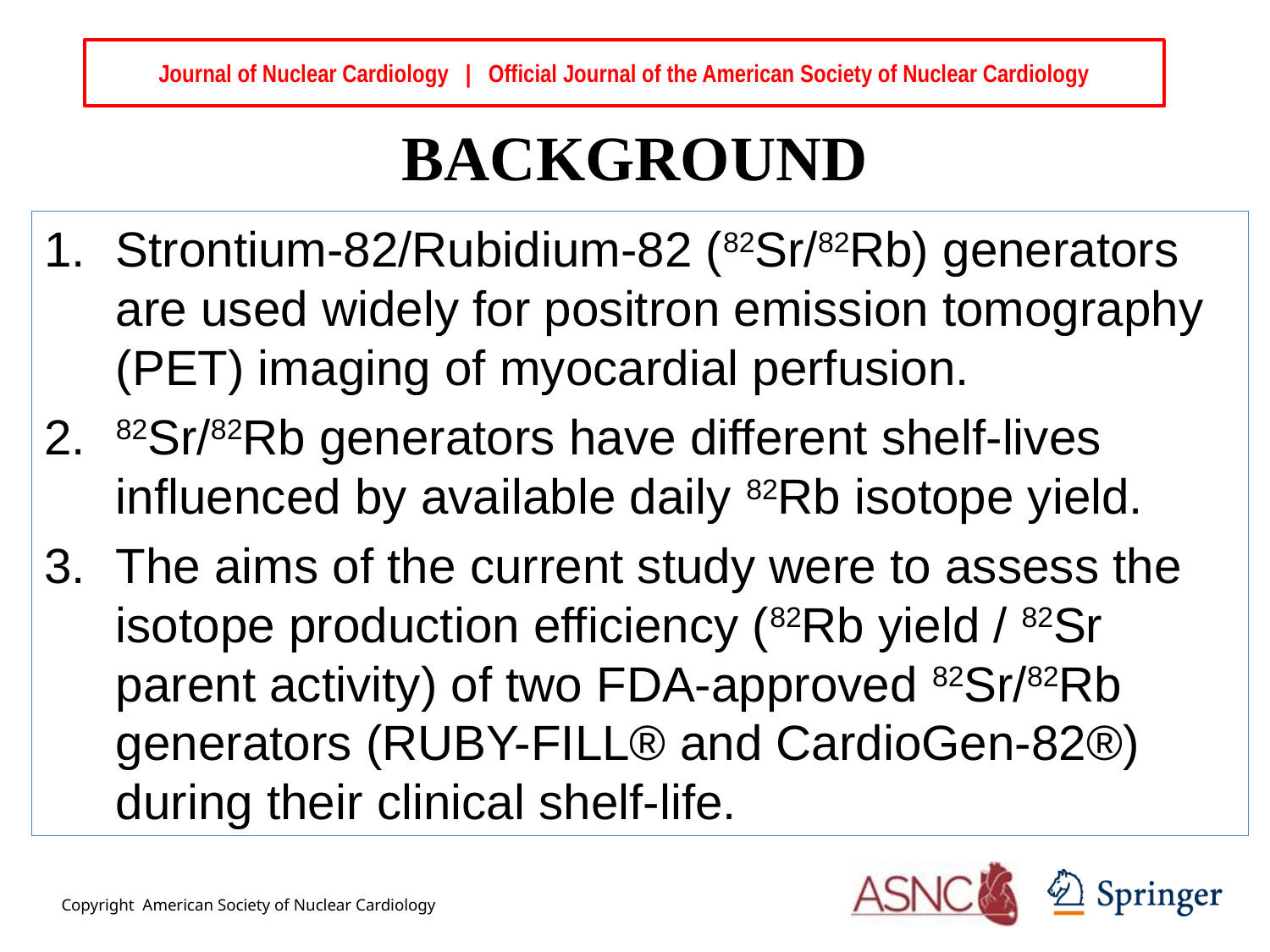

Journal of Nuclear Cardiology | Official Journal of the American Society of Nuclear Cardiology
# BACKGROUND
Strontium-82/Rubidium-82 (82Sr/82Rb) generators are used widely for positron emission tomography (PET) imaging of myocardial perfusion.
82Sr/82Rb generators have different shelf-lives influenced by available daily 82Rb isotope yield.
The aims of the current study were to assess the isotope production efficiency (82Rb yield / 82Sr parent activity) of two FDA-approved 82Sr/82Rb generators (RUBY-FILL® and CardioGen-82®) during their clinical shelf-life.
Copyright American Society of Nuclear Cardiology

## Slide 3
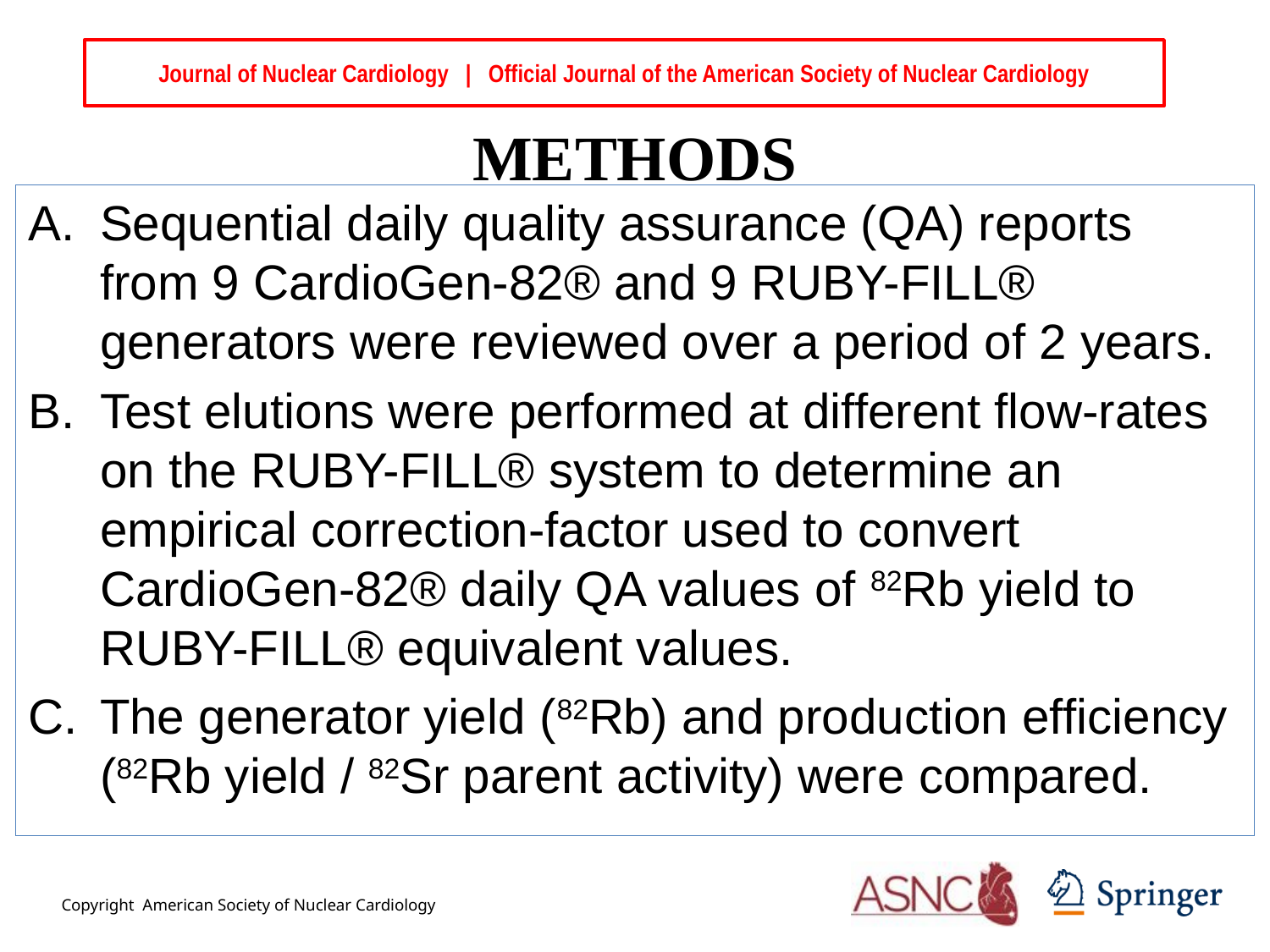

Journal of Nuclear Cardiology | Official Journal of the American Society of Nuclear Cardiology
# METHODS
Sequential daily quality assurance (QA) reports from 9 CardioGen-82® and 9 RUBY-FILL® generators were reviewed over a period of 2 years.
Test elutions were performed at different flow-rates on the RUBY-FILL® system to determine an empirical correction-factor used to convert CardioGen-82® daily QA values of 82Rb yield to RUBY-FILL® equivalent values.
The generator yield (82Rb) and production efficiency (82Rb yield / 82Sr parent activity) were compared.
Copyright American Society of Nuclear Cardiology

## Slide 4
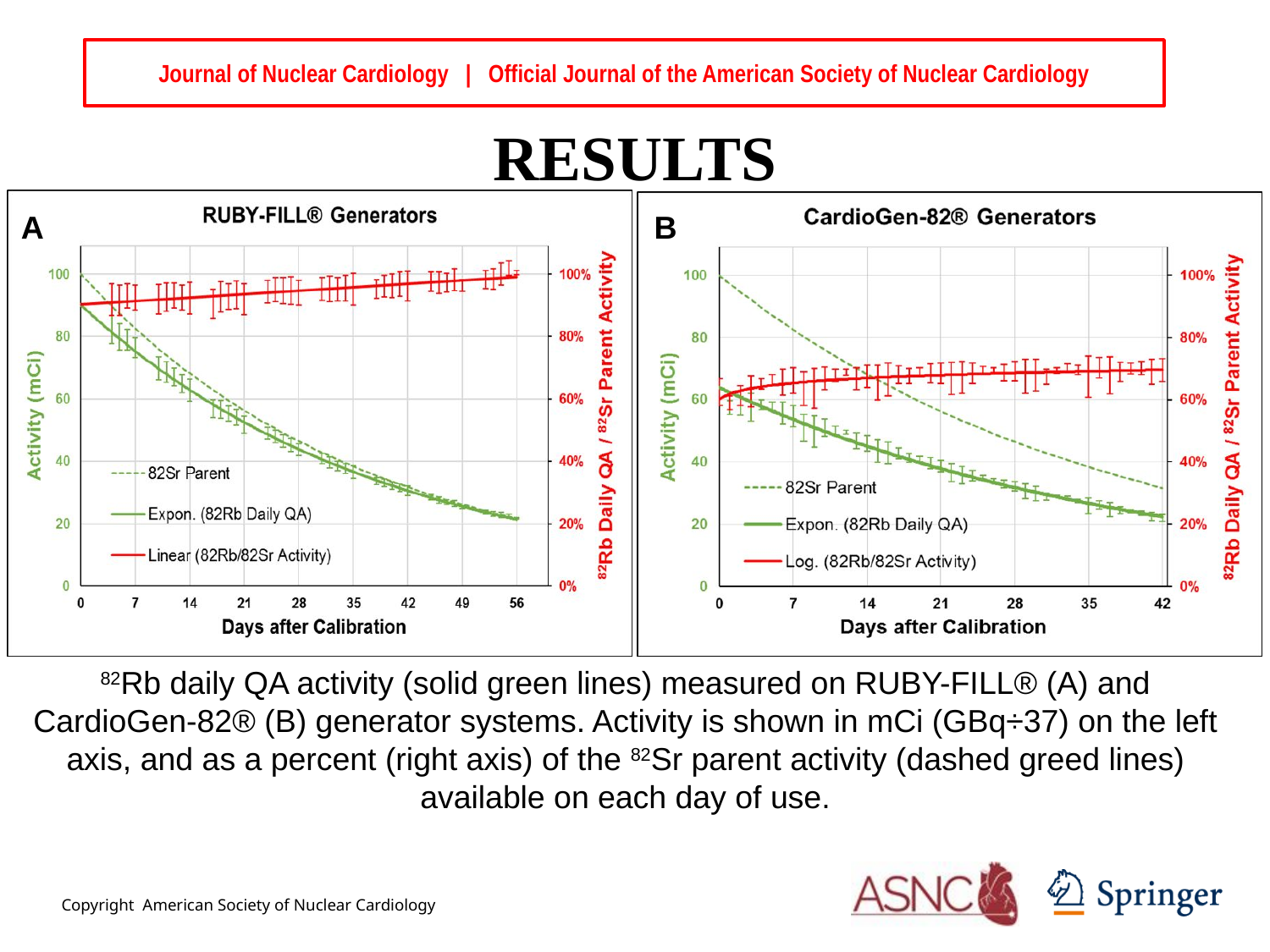

Journal of Nuclear Cardiology | Official Journal of the American Society of Nuclear Cardiology
# RESULTS
A
B
82Rb daily QA activity (solid green lines) measured on RUBY-FILL® (A) and
CardioGen-82® (B) generator systems. Activity is shown in mCi (GBq÷37) on the left
axis, and as a percent (right axis) of the 82Sr parent activity (dashed greed lines)
available on each day of use.
Copyright American Society of Nuclear Cardiology

## Slide 5
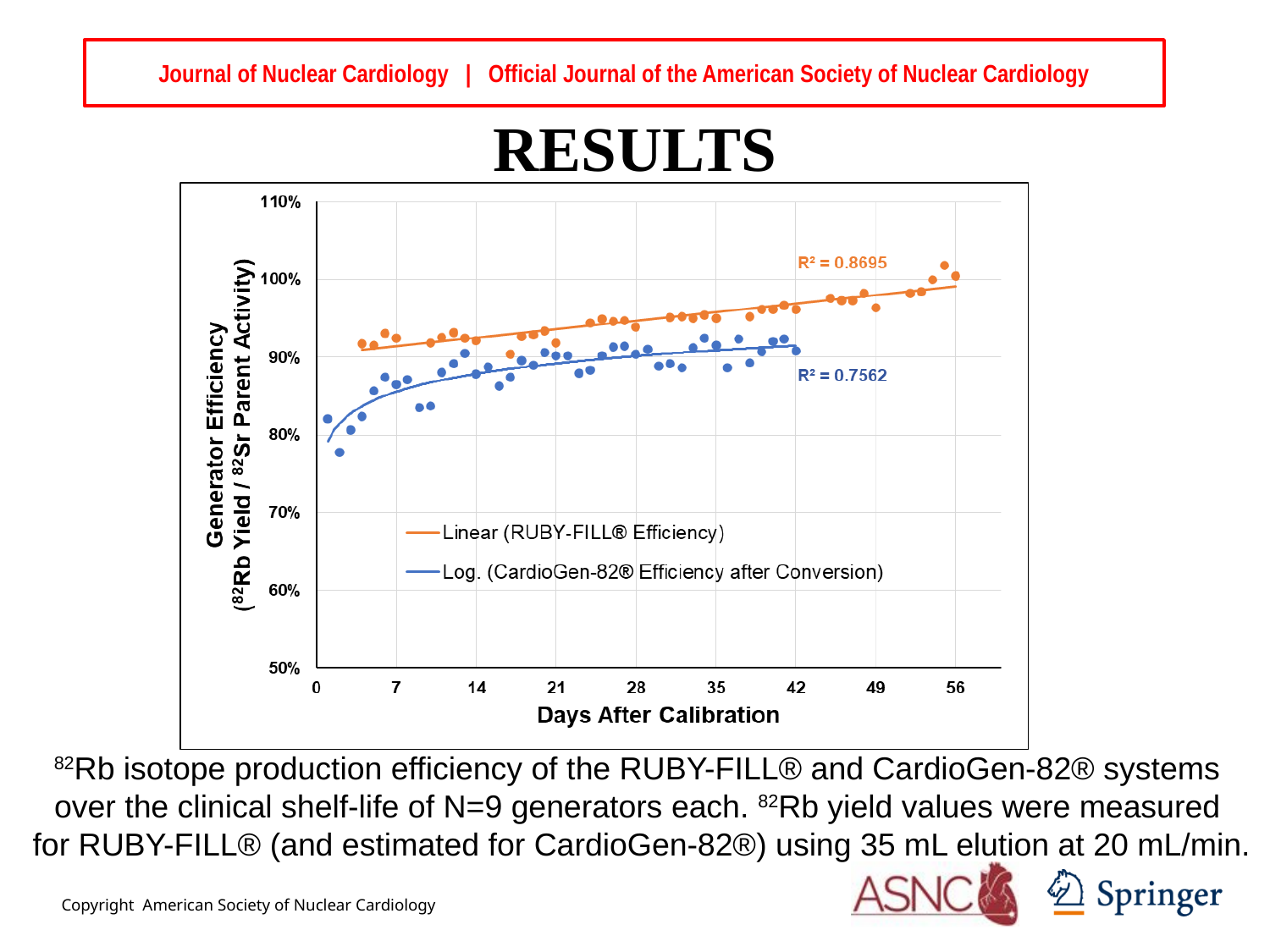

Journal of Nuclear Cardiology | Official Journal of the American Society of Nuclear Cardiology
# RESULTS
82Rb isotope production efficiency of the RUBY-FILL® and CardioGen-82® systems
over the clinical shelf-life of N=9 generators each. 82Rb yield values were measured
for RUBY-FILL® (and estimated for CardioGen-82®) using 35 mL elution at 20 mL/min.
Copyright American Society of Nuclear Cardiology

## Slide 6
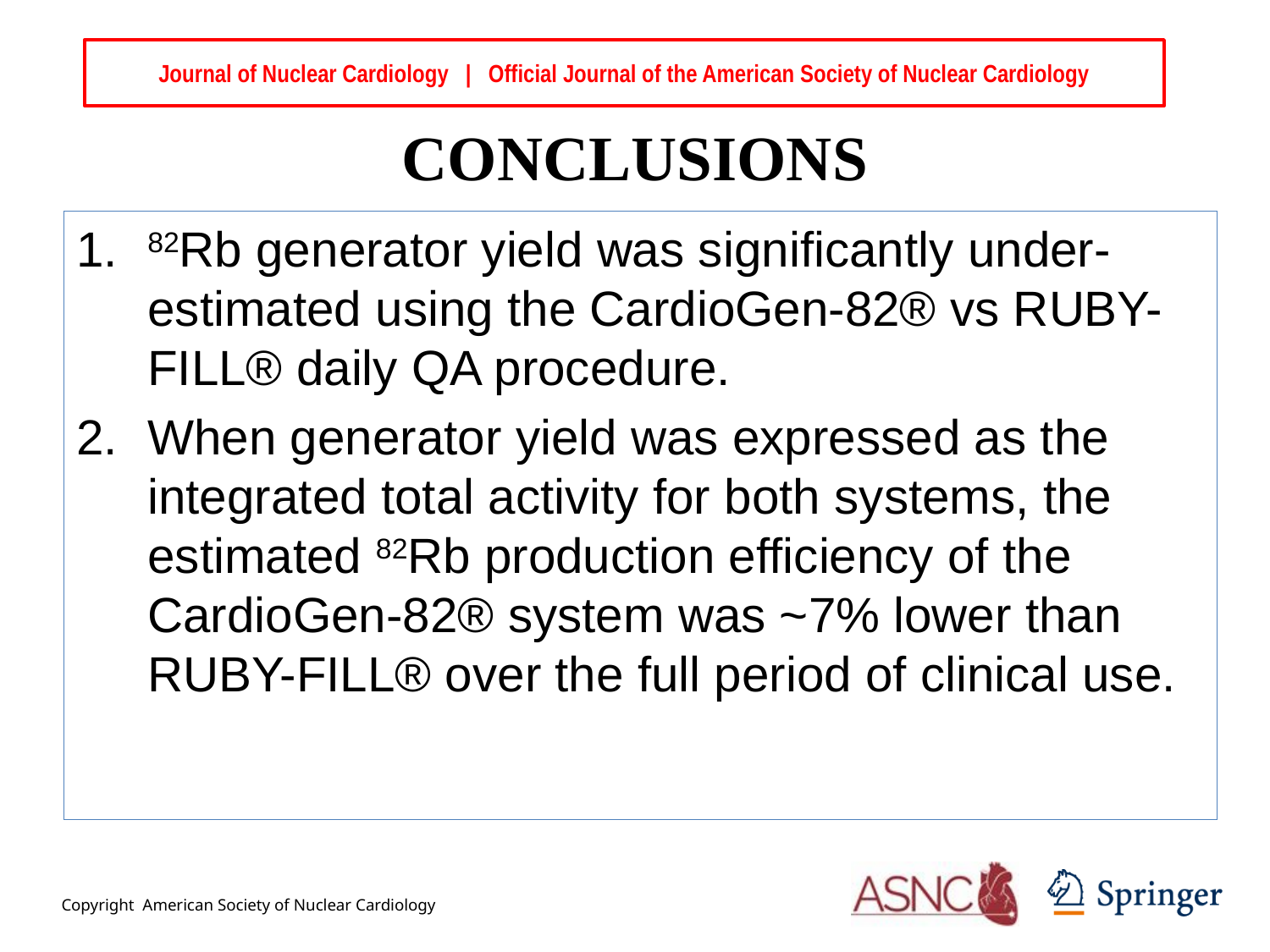

Journal of Nuclear Cardiology | Official Journal of the American Society of Nuclear Cardiology
# CONCLUSIONS
82Rb generator yield was significantly under-estimated using the CardioGen-82® vs RUBY-FILL® daily QA procedure.
When generator yield was expressed as the integrated total activity for both systems, the estimated 82Rb production efficiency of the CardioGen-82® system was ~7% lower than RUBY-FILL® over the full period of clinical use.
Copyright American Society of Nuclear Cardiology
